# Mapache: a flexible pipeline to map ancient DNA

**DOI:** 10.1093/bioinformatics/btad028

**Published:** 2023-01-13

**Authors:** Samuel Neuenschwander, Diana I Cruz Dávalos, Lucas Anchieri, Bárbara Sousa da Mota, Davide Bozzi, Simone Rubinacci, Olivier Delaneau, Simon Rasmussen, Anna-Sapfo Malaspinas

**Affiliations:** Department of Computational Biology, University of Lausanne, Lausanne 1015, Switzerland; Vital-IT, Swiss Institute of Bioinformatics, Lausanne 1015, Switzerland; Department of Computational Biology, University of Lausanne, Lausanne 1015, Switzerland; Swiss Institute of Bioinformatics, Lausanne 1015, Switzerland; Department of Computational Biology, University of Lausanne, Lausanne 1015, Switzerland; Swiss Institute of Bioinformatics, Lausanne 1015, Switzerland; Department of Computational Biology, University of Lausanne, Lausanne 1015, Switzerland; Swiss Institute of Bioinformatics, Lausanne 1015, Switzerland; Department of Computational Biology, University of Lausanne, Lausanne 1015, Switzerland; Swiss Institute of Bioinformatics, Lausanne 1015, Switzerland; Department of Computational Biology, University of Lausanne, Lausanne 1015, Switzerland; Swiss Institute of Bioinformatics, Lausanne 1015, Switzerland; Department of Computational Biology, University of Lausanne, Lausanne 1015, Switzerland; Swiss Institute of Bioinformatics, Lausanne 1015, Switzerland; Novo Nordisk Foundation Center for Protein Research, University of Copenhagen, Copenhagen 2200, Denmark; Department of Computational Biology, University of Lausanne, Lausanne 1015, Switzerland; Swiss Institute of Bioinformatics, Lausanne 1015, Switzerland

## Abstract

**Summary:**

We introduce mapache, a flexible, robust and scalable pipeline to map, quantify and impute ancient and present-day DNA in a reproducible way. Mapache is implemented in the workflow manager Snakemake and is optimized for low-space consumption, allowing to efficiently (re)map large datasets—such as reference panels and multiple extracts and libraries per sample — to one or several genomes. Mapache can easily be customized or combined with other Snakemake tools.

**Availability and implementation:**

Mapache is freely available on GitHub (https://github.com/sneuensc/mapache). An extensive manual is provided at https://github.com/sneuensc/mapache/wiki.

**Supplementary information:**

Supplementary data are available at *Bioinformatics* online.

## 1 Introduction

Mapping sequencing reads to a reference genome is a fundamental step in genomic analyses. Compared with modern data, ancient DNA (aDNA) presents a number of challenges as data are sparse, contaminated and affected by post-mortem damage, such as fragmentation and deamination. Furthermore, samples are often re-sequenced many times to maximize their yield, making the mapping step iterative. As a result, robust, efficient, yet easy to use bioinformatic pipelines are needed to map and remap the sequencing reads in a reproducible way, while allowing a wide range of users to modify predefined options and navigate through the results to, for example, select promising samples, extracts or libraries. Furthermore, intermediate files might require one order of magnitude more of storage compared with the final BAM files, thus researchers need efficient pipelines since storage is costly and space quotas can be quickly reached during mapping. For example, mapping 8.5 TB of FASTQ files (Harris *et al.*, 2018) would require about 140 TB of storage for temporary files, while the final mapped files measure only 9.9 TB.

While PALEOMIX (based on Python; Schubert *et al.*, 2014) and nf-core/eager (based on Nextflow/Groovy; Yates *et al.*, 2021) have emerged as the main pipelines to map aDNA, both require considerable amounts of storage for temporary files, may not be executed on modern clusters with queuing systems (PALEOMIX) or are limited to a single reference genome (nf-core/eager).

Here, we introduce mapache, a lightweight pipeline designed to map both ancient and present-day DNA sequences to one or multiple reference genomes while generating reports with summary statistics to inspect the quality of the mappings. Mapache is implemented in the workflow manager Snakemake (Mölder *et al.*, 2021) and it thus inherits its flexibility, scalability and reproducibility. Snakemake is based on Python allowing users to easily adapt the code or to combine the workflow with other Snakemake workflows. Moreover, unlike the other tools, mapache efficiently manages temporary files, keeping the necessary storage space small. Furthermore, mapache can map sequences to more than one genome in parallel and the pipeline can be used on modern clusters with queuing systems. Finally, mapache has an additional module to allow for genotype imputation of low-coverage genomes with GLIMPSE (Rubinacci *et al.*, 2021), a step that may become quite common in aDNA (see e.g. Sousa da Mota *et al.*, 2022).

## 2 Approach and features

### 2.1 Scalability

**Fig. 1.**
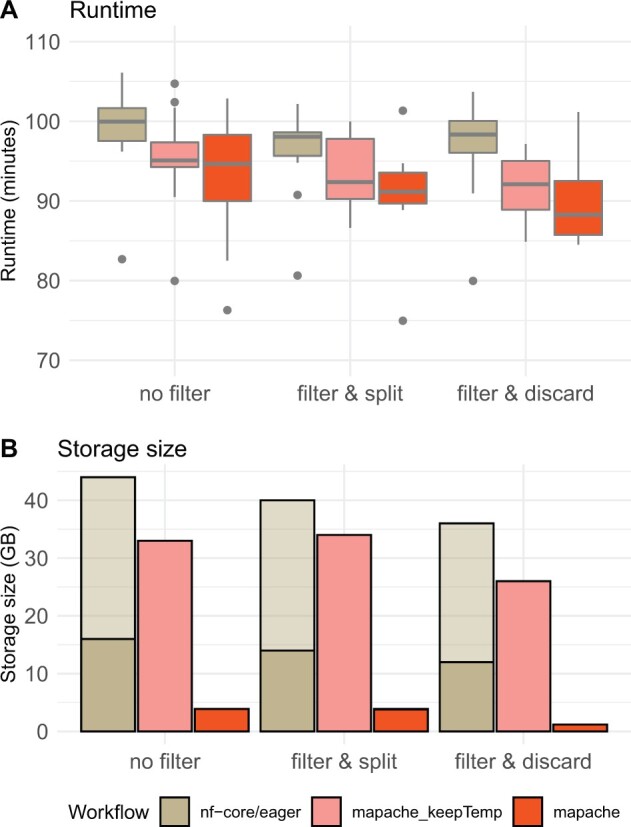
Viking-age genomic data of cod were mapped. To make the runs comparable, the settings of mapache and nf-core/eager were adapted so that the two pipelines are as similar as possible. Mapache was run in two modes: “mapache_keepTemp”; keeping all intermediate files (snakemake parameter --nt) - similar to nf-core/eager - and “mapache”; removing all intermediate files and minimizing the storage space (default behavior of mapache). The outputs vary across runs: 1) “no filter”: no quality filtering applied (BAM file includes all data); 2) “filter & split”: one BAM file with high-quality alignments and one with low-quality alignments and/or unmapped reads; 3) “filter & discard”: storing only one BAM file containing only high-quality alignments. Each individual configuration was run 15 times on a single machine with 32 CPUs (AMD EPYC 7443) and 256 GB of memory. See Supplementary File for details. A) Runtime in minutes. B) Storage size at the end of a run. nf-core/eager stores the data in two folders, folder ‘results’ contains the main output (dark color) and folder ‘work’ contains the intermediate files (light color).

Mapache scales well for different research cases: screening for aDNA quality (mapping of many small FASTQ files), mapping of high-coverage genomes (many large FASTQ files) to a single reference as well as reconstructing microbial genomes from a metagenomic sample (mapping many FASTQ files to many reference genomes). Depending on the input size, mapache can be launched locally or on a distributed system. Mapache can continuously delete temporary files to reduce the space required on the file system. Our benchmark on a dataset of ∼167 million reads shows that mapache requires considerably less storage space for the final outputs (3.7 GB versus 44 GB) and that it runs faster than nf-core/eager (Figure 1; see Supplementary File for details and discussion on the benchmarking). Mapache’s final output in the benchmark example (3.7 GB) is composed mainly by two BAM files per sample, one with mapped reads (1.1 GB) and another one with low-quality and unmapped reads (1.6 GB), and mapping statistics (64 MB). In mapache’s case, a total of 30 GB of intermediate files (e.g. trimmed reads, intermediate unsorted BAMs and unmerged BAMs, etc.) were deleted as soon as they were no longer needed.

Finally, as mapache can be run on a cluster and since it efficiently handles the storage, it can be used on large-scale datasets. For instance, the mapping of six high-coverage ancient human genomes (246 GB, 743 million reads; Clemente *et al.*, 2021) took 2.5 h and the mapping of 150 modern human genomes (8.5 TB of input data; Harris *et al.*, 2018, European Genome-Phenome Archive: EGAD00001007082) took less than a week on a cluster.

### 2.2 Automation

Prior to run mapache, the user has to prepare a plain text file listing the input FASTQ files and their metadata (e.g. sample and library names). The amount of memory and running time needed for each step can be specified through a configuration file, as well as parameters specific to the bioinformatic tools included in the pipeline. Mapache can then be launched with a single command line.

As mentioned above, intermediate files can be kept or removed (default) once they are no longer needed. Therefore, if the pipeline fails at any step (e.g. due to a lack of memory or time allocated), it can be re-launched with the same command. This would trigger the re-execution of the failed steps and other pending analyses; that is, successful tasks will not be rerun and their outputs will be automatically used in the next steps when needed.

### 2.3 Portability

Mapache can easily be installed on any Linux computer. Dependencies are automatically installed using Conda.

## 3 Workflow

### 3.1 Configuration file

Mapache can be customized using a configuration file. The configuration is highly flexible, allowing the user to adapt mapache to the input data: (1) steps of the workflow can be skipped, (2) several softwares are available for given steps and (3) any tool-specific parameters may be included in the pipeline. For example, the mapping pipeline can be customized with parameters suited to map present-day DNA; the user can select among BWA-aln, BWA-mem and bowtie2 for mapping; any valid parameters can be passed to the individual tools included in the pipeline. Finally, the workflow can be adapted to the available computer infrastructure by specifying the number of threads, amount of memory and running time for each step in the configuration file.

### 3.2 Mapping workflow

**Fig. 2.**
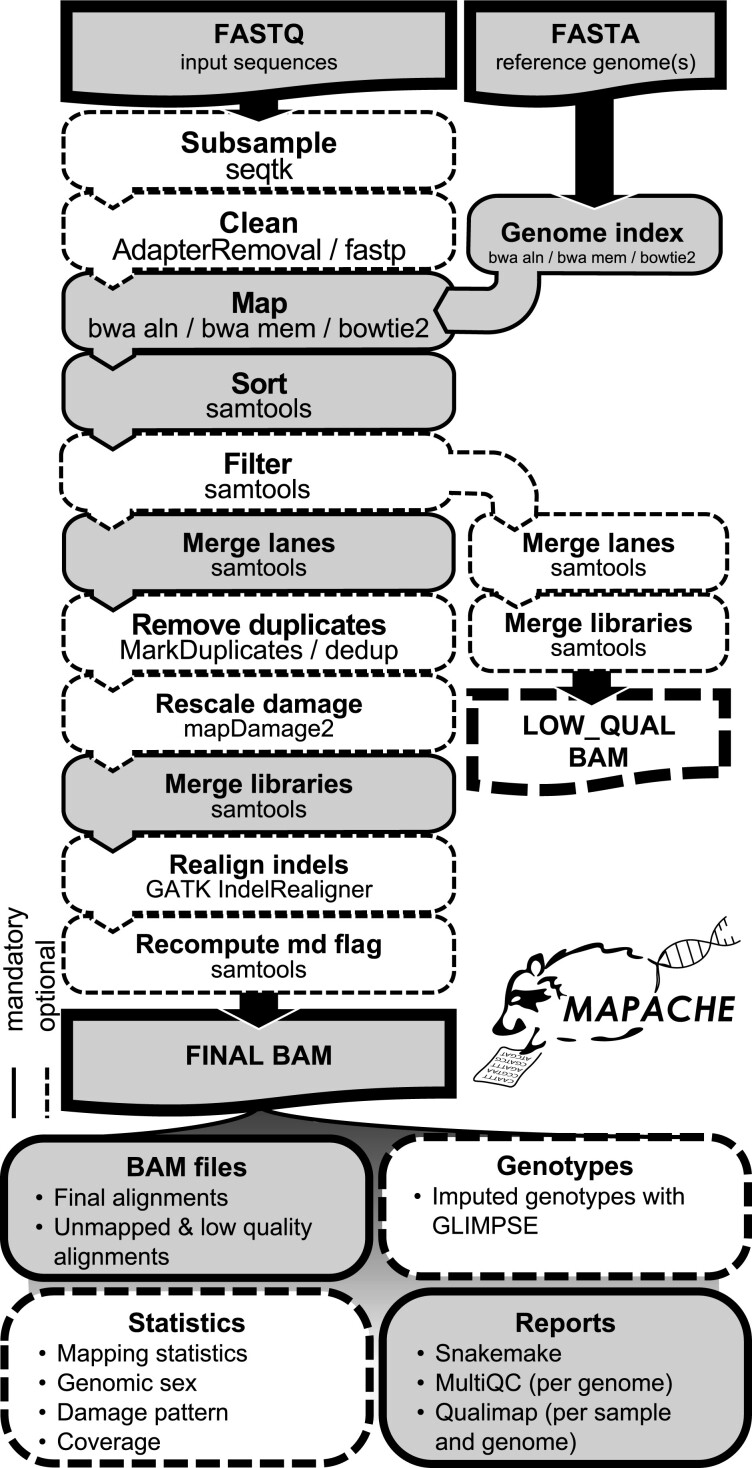
Main workflow of mapache. Each box shows a step and related software. Dashed/white boxes are optional

A detailed description of all the steps of the workflow can be found on the wiki. A diagram of the main steps is shown in Figure 2. Mapache is optimized for mapping aDNA reads to the human reference genome. In brief, adapters, short reads and low-quality bases can be removed with AdapterRemoval2 (Schubert *et al.*, 2016, run by default) or fastp (Chen *et al.*, 2018). Reads are mapped with BWA aln or mem (Li and Durbin, 2009, aln run by default) or with Bowtie2 (Langmead and Salzberg, 2012) and merged by library. Duplicates can be removed per library with Picard MarkDuplicates (https://broadinstitute.github.io/picard/, run by default) or dedup (Peltzer *et al.*, 2016), and libraries are merged by sample. Finally, reads can be realigned locally around indels with GATK (DePristo *et al.*, 2011) and the MD flag can be recomputed with SAMtools (Danecek *et al.*, 2021). Unmapped and low-quality mapped reads can be stored in a separate BAM file per sample.

### 3.3 Imputation (optional)

Mapache allows for imputation and phasing using GLIMPSE, a tool that is shown to accurately impute both low-coverage present-day (Rubinacci *et al.*, 2021) and ancient human genomes (Sousa da Mota *et al.*, 2022).

### 3.4 Output

Mapache’s main outputs are the alignments to the reference genomes as BAM files and, if requested, (a) low-quality alignments and unmapped reads and (b) imputed genotypes in VCF format. Mapache outputs summary statistics on the final BAM and intermediate files, including depth of coverage for all or a subset of the chromosomes (Quinlan and Hall, 2010), genomic sex, damage patterns (Jónsson *et al.*, 2013; Malaspinas *et al.*, 2014) and read lengths. Statistics are returned as tables and graphs, allowing to quickly investigate the results. For in-depth analyses, mapache can generate several individual reports (fastqc, Andrews, 2010; qualimap, Okonechnikov *et al.*, 2016; multiqc, Ewels *et al.*, 2016). At the end of a run, mapache will output a plain-text file with the versions of the software used. A self-contained html report with all graphs and tables can be generated in a single command at the end of a run. The summary statistics computation and the imputation can also be run for any BAM file not generated by mapache.

## 4 Conclusion

Mapache is a flexible mapping workflow optimized for runtime and minimal storage usage to efficiently map both ancient and present-day DNA sequences in a reproducible manner. Mapache comes with an extensive wiki and pre-defined parameters to facilitate its use. Furthermore, as the underlying language is Python, users may modify the code or combine mapache with any other Snakemake workflow. Finally, mapache includes downstream processes that are also standalone and can be run on existing BAM files.

## Supplementary Material

btad028_Supplementary_Data
