# Supplementary material for: Mapache: a flexible pipeline to map ancient DNA
Source: Bioinformatics. 2023 Jan 13;39(2):btad028. doi: 10.1093/bioinformatics/btad028 (PMC9901408; doi:10.1093/bioinformatics/btad028)
Supplement: btad028_Supplementary_Data [file btad028_supplementary_data.zip › Supplementary_software.pdf]

Supplementary Information: Software

The following table lists all software used for the different steps of the workflow.

	Version	Reference	Link
**Workflow manager**			
Snakemake	7.18.2	Mölder, et al., 2021	https://github.com/snakemake/snakemake
**Subsample**			
seqtk	1.3		https://github.com/lh3/seqtk
**Clean**			
AdapterRemoval2	2.3.2	Schubert, et al., 2016	https://github.com/MikkelSchubert/adapterremoval
fastp	0.23.2	Chen, et al., 2018	https://github.com/OpenGene/fastp
**Map**			
BWA aln	0.7.17	Li and Durbin, 2010	https://github.com/lh3/bwa
BWA mem	0.7.17	Li and Durbin, 2010	https://github.com/lh3/bwa
Bowtie2	2.4.4	Langmead and Salzberg, 2012	https://github.com/BenLangmead/bowtie2
**Sort**			
SAMtools	1.14	Danecek, et al., 2021	https://github.com/samtools/samtools
**Filter**			
SAMtools	1.14	Danecek, et al., 2021	https://github.com/samtools/samtools
**Merge lanes**			
SAMtools	1.14	Danecek, et al., 2021	https://github.com/samtools/samtools
**Remove duplicates**			
Picard MarkDuplicates	2.25.5	Broad Institute, 2019	http://broadinstitute.github.io/picard
dedup	0.12.8	Peltzer, et al., 2016	https://github.com/apeltzer/DeDup
**Rescale damage**			
mapDamage2	2.2.1	Jonsson, et al., 2013	https://github.com/ginolhac/mapDamage
**Merge libraries**			
SAMtools	1.14	Danecek, et al., 2021	https://github.com/samtools/samtools
**Realign indels**			
GATK IndelRealigner	3.8	DePristo, et al., 2011	https://gatk.broadinstitute.org
**Recompute md flag**			
SAMtools	1.14	Danecek, et al., 2021	https://github.com/samtools/samtools
**Imputation**			
GLIMPSE	1.1.1	Rubinacci, et al., 2021	https://github.com/odelaneau/GLIMPSE
BCFtools	1.15	Danecek, et al., 2021	https://github.com/samtools/bcftools
**Reports**			
FastQC	0.11.9	Andrews, 2010	https://www.bioinformatics.babraham.ac.uk/projects/fastqc
Qualimap	2.2.2d	Okonechnikov, et al., 2016	http://qualimap.conesalab.org
MultiQC	1.13	Ewels, et al., 2016	https://multiqc.info
**Statistics**			
BEDTools	2.30.0	Quinlan and Hall, 2010	https://github.com/arq5x/bedtools2
bamdamage	modified	Malaspinas, et al., 2014	https://savannah.nongnu.org/projects/bammds
R	4.0	R Core Team, 2022	https://www.r-project.org

References

Picard tools. In, *Broad Institute, GitHub repository*. http://broadinstitute.github.io/picard: Broad Institute; 2019.

Andrews, S. FASTQC. A quality control tool for high throughput sequence data. In.; 2010.

Chen, S., *et al.* fastp: an ultra-fast all-in-one FASTQ preprocessor. *Bioinformatics* 2018;34(17):i884-i890.

Danecek, P., *et al.* Twelve years of SAMtools and BCFtools. *GigaScience* 2021;10(2).

DePristo, M.A., *et al.* A framework for variation discovery and genotyping using next-generation DNA sequencing data. *Nature Genetics* 2011;43(5):491-+.

Ewels, P., *et al.* MultiQC: summarize analysis results for multiple tools and samples in a single report. *Bioinformatics* 2016;32(19):3047-3048.

Jonsson, H., *et al.* mapDamage2.0: fast approximate Bayesian estimates of ancient DNA damage parameters. *Bioinformatics* 2013;29(13):1682-1684.

Langmead, B. and Salzberg, S.L. Fast gapped-read alignment with Bowtie 2. *Nat Methods* 2012;9(4):357-359.

Li, H. and Durbin, R. Fast and accurate long-read alignment with Burrows-Wheeler transform. *Bioinformatics* 2010;26(5):589-595.

Malaspinas, A.S., *et al.* bammds: a tool for assessing the ancestry of low-depth whole-genome data using multidimensional scaling (MDS). *Bioinformatics* 2014;30(20):2962-2964.

Mölder, F., *et al.* Sustainable data analysis with Snakemake [version 1; peer review: awaiting peer review]. *F1000Research* 2021;10(33).

Okonechnikov, K., Conesa, A. and Garcia-Alcalde, F. Qualimap 2: advanced multi-sample quality control for high-throughput sequencing data. *Bioinformatics* 2016;32(2):292-294.

Peltzer, A., *et al.* EAGER: efficient ancient genome reconstruction. *Genome Biology* 2016;17.

Quinlan, A.R. and Hall, I.M. BEDTools: a flexible suite of utilities for comparing genomic features. *Bioinformatics* 2010;26(6):841-842.

R Core Team. 2022. R: A Language and Environment for Statistical Computing. http://www.R-project.org

Rubinacci, S., *et al.* Efficient phasing and imputation of low-coverage sequencing data using large reference panels. *Nature Genetics* 2021;53(1):120-126.

Schubert, M., Lindgreen, S. and Orlando, L. AdapterRemoval v2: rapid adapter trimming, identification, and read merging. *BMC Res Notes* 2016;9:88.
