# Supplementary material for: Mapache: a flexible pipeline to map ancient DNA
Source: Bioinformatics. 2023 Jan 13;39(2):btad028. doi: 10.1093/bioinformatics/btad028 (PMC9901408; doi:10.1093/bioinformatics/btad028)
Supplement: btad028_Supplementary_Data [file btad028_supplementary_data.zip › Supplementary_benchmarking[AU].pdf]

Supplementary Information: Benchmark

To evaluate the performance of mapache compared to previously published ancient DNA mapping pipelines, we mapped two samples of Viking-age genomic data of cod (*Gadus morhua*) from (Star, et al., 2017) to the reference genome CAEA02 (Tørresen, et al., 2017). Note that the cod dataset was already used to benchmark nf-core/eager (Yates, et al., 2021) and the two samples (COD076 and COD092) consist each of three Illumina paired-end libraries with ~167 million reads in total. We used both mapache and nf-core/eager with different settings for this analysis.

Although PALEOMIX (Schubert, et al., 2014) could be run on a cluster with a queuing system (single job submission on a single multithreaded machine), it is not possible to distribute its individual processes across multiple nodes, and thus to take advantage of a modern cluster with hundreds or thousands of nodes. In practical terms, the user would be quickly limited in the number of inputs that can be processed simultaneously as the size of the project increases. Thus, as PALEOMIX was not designed to be deployed on machines/a cluster with a queuing system, we did not include it in this benchmarking.

To make the benchmarking comparable we adapted the settings of mapache and nf-core/eager so that the two pipelines are as similar as possible (see below for the exact settings). The mapping workflow consisted in removing adapters and collapsing pairs of reads with AdapterRemoval2 (parameters --collapse --minlength 30 --trimns --trimqualities --minquality 20 --adapter1 AGATCGGAAGAGCACACGTCTGAACTCCAGTCACNNNNNNNATCTCGTATGCCGTC TTCTGCTTG; Schubert, et al. (2016)), mapping with BWA aln (parameters -n 0.04 -o 1 -k 2 -l 1024) and BWA samse (Li and Durbin, 2010), sorting BAM file with samtools (Danecek, et al., 2021), removing duplicates with Picard's MarkDuplicates (Broad Institute, (2019), and merging libraries with samtools (Danecek, et al., 2021).

The resulting alignment files were very similar and only differed due to stochastic processes in AdapterRemoval's "collapsing step" (while AdapterRemoval outputs the same number of reads, the sequences themselves are not identical; Table 1). Note, however, that, by design, other outputs of the workflows differ. For instance, after mapping, both pipelines filtered the alignment file for mapping quality and stored the high-quality alignments in the main alignment file. In mapache, the low-quality alignments and unmapped reads are stored in a separate alignment file, while nf-core/eager discards low-quality alignments and stores only the unmapped reads in a separate alignment file. The two workflows differ also in the summary statistics computed along the run. For example, damage profiles are investigated with a modified version of bamdamage (Malaspinas, et al., 2014) and with DamageProfiler (Neukamm, et al., 2021) for mapache and nf-core/eager, respectively.

To obtain a better understanding on the runtime and required space, we ran mapache in two different modes:

- *mapache_keepTemp*: keeping all intermediate files, similar to nf-core/eager (snakemake parameter --nt)
- *mapache*: removing all intermediate files and minimizing the storage space (default behavior of mapache)

We further investigated the runtimes and space requirements for each workflow. We considered the following setups in terms of how the low-quality and unmapped reads are handled by mapache and nf-core/eager:

- *no filter*: the final BAM files contain all the alignments: high-quality, low-quality and unmapped ones (no quality filtering applied).
- *filter & split*: there are two final BAM files as output, one containing high-quality alignments (samtools view -F 4 -q 30; Danecek, et al. (2021)), and another BAM file with either
    - low-quality alignments and unmapped reads (mapache)
    - unmapped reads (nf-core/eager).
- *filter & discard*: storing only one BAM file containing only high-quality alignments (samtools view -F 4 -q 30; Danecek, et al. (2021)).

Each individual configuration was run 15 times on a single machine with 32 CPUs (AMD EPYC 7443) and 256 GB of memory.

Results & Discussion

A priori, we expected small differences in runtimes between mapache and nf-core/eager, as the underlying computationally intensive tools are identical. In this analysis, mapache had shorter running times than nf-core/eager (Figure 1A). Differences may be explained by different actions on intermediate outputs (e.g., scripts to compute statistics), some of which cannot be skipped by the user but nevertheless contribute to the total running times, or the implementation of the workflow managers.

Mapache uses considerably less storage space than nf-core/eager (Figure 1B) when all intermediate files are kept (*nf-core/eager* versus *mapache_keepTemp*). The default run with mapache (removing all intermediate files when they are no longer needed) uses only a fraction of the disk space compared to the runs keeping all files.

nf-core/eager stores files in two folders, the 'results' folder containing the main files, and the 'work' folder containing all intermediate files (dark and light color in Figure 1B, respectively). The 'work' folder can be removed after the run, significantly reducing the space. However, the

remaining space is still considerably higher than the space used by the default run with mapache (*mapache* bar in Figure 1B). Moreover, the space can only be freed after the run has terminated, requiring enough space on the disk to accommodate the temporary files. In contrast, mapache removes the temporary files as soon as they are no longer needed, avoiding any space usage peak.

For instance, for the run with mapache that requires the largest amount of storage (34G, mapache_keepTemp + no filter, first pink bar in Fig. 1B), the space is freed along the run (mapache + no filter, first orange bar in Fig. 1B) as follows: the reference genome index (1.1G) and the trimmed reads (8G) are erased once all input FASTQ files have been mapped. The resulting SAI and BAM files (7.7G) are removed once the BAM file is sorted (3.9G). This sorted BAM file (3.9G) is erased after splitting the reads into unmapped and low-quality reads (2.7G), and high-quality reads (1.2G). The intermediate BAMs containing unmapped and low-quality reads for every inputted FASTQ file are removed after being merged into their corresponding BAM per library (2.6G). Similarly, the intermediate high-quality BAM files are erased after generating a BAM file without duplicates (1.3G). At this point, there are as many BAM files with high-quality reads as libraries in the dataset. The library BAM files are then removed after being merged into a corresponding sample BAM file (1.1G). Likewise, the BAM files with unmapped and low-quality reads per library are merged (and then removed) into the corresponding sample BAM files (2.6G). Therefore, the run with mapache + no filter contains only 3.7G, corresponding mainly to: (i) the final BAM files per sample with unmapped and low- (2.6G) and high-quality reads (1.1G) and (ii) the reports and statistics, which are not erased as they occupy significantly less space (64 MB). Finally, since the temporary files are removed only after they are no longer needed, in the event of a failed run (e.g., due to lack of memory or run time), mapache will resume the pipeline starting from the failed step (i.e., there is no need to re-execute previous successful steps).

Note that, for mapache, the smallest space requirements are achieved when discarding the low-quality alignments and unmapped reads. This is ideal when (re)mapping large amounts of data and in cases where only the high-quality alignments are needed. When starting to work with new ancient samples, it may be preferable to keep all intermediate files to be able to investigate them and/or incrementally add new sequencing data.

Table 1. List of number of reads/alignments after the different steps of benchmark runs. The stochasticity comes from AdapterRemoval's collapsing. The table shows two randomly selected runs. 'diff-abs' shows the absolute difference and 'diff-%' shows the relative difference.

sample	library	**eager**	**mapache**	**diff-abs**	**diff-%**
**fastq original**					
	ERR1943600	13,154,136			
COD076	ERR1943601	40,517,845			
	ERR1943602	23,271,181			
	ERR1943607	30,165,251			
COD092	ERR1943608	30,114,584			
	ERR1943609	30,075,651			
**fastq after adapter removal**					
	ERR1943600	11,810,838	11,810,838	0	0%
COD076	ERR1943601	36,448,705	36,448,705	0	0%
	ERR1943602	20,906,714	20,906,714	0	0%
	ERR1943607	21,672,528	21,672,528	0	0%
COD092	ERR1943608	21,629,669	21,629,669	0	0%
	ERR1943609	21,569,152	21,569,152	0	0%
**bam after mapping**					
	ERR1943600	11,810,838	11,810,838	0	0%
COD076	ERR1943601	36,448,705	36,448,705	0	0%
	ERR1943602	20,906,714	20,906,714	0	0%
	ERR1943607	21,672,528	21,672,528	0	0%
COD092	ERR1943608	21,629,669	21,629,669	0	0%
	ERR1943609	21,569,152	21,569,152	0	0%
**bam after filtering**					
	ERR1943600	2,902,089	2,901,994	95	0.003%
COD076	ERR1943601	8,793,504	8,793,548	-44	-0.001%
	ERR1943602	4,990,614	4,990,623	-9	0.000%
	ERR1943607	5,598,657	5,598,761	-104	-0.002%
COD092	ERR1943608	5,588,007	5,588,051	-44	-0.001%
	ERR1943609	5,579,468	5,579,522	-54	-0.001%
**bam after duplicates removal**					
	COD076_lib1	2,704,948	2,704,853	95	0.004%
COD076	COD076_lib2	7,274,496	7,274,502	-6	0.000%
	COD076_lib3	4,448,045	4,448,121	-76	-0.002%
	COD092_lib1	5,247,078	5,247,189	-111	-0.002%
COD092	COD092_lib2	5,238,242	5,238,301	-59	-0.001%
	COD092_lib3	5,231,511	5,231,562	-51	-0.001%
**bam final**					
COD076		14,427,489	14,427,476	13	0.000%
COD092		15,716,831	15,717,052	-221	-0.001%

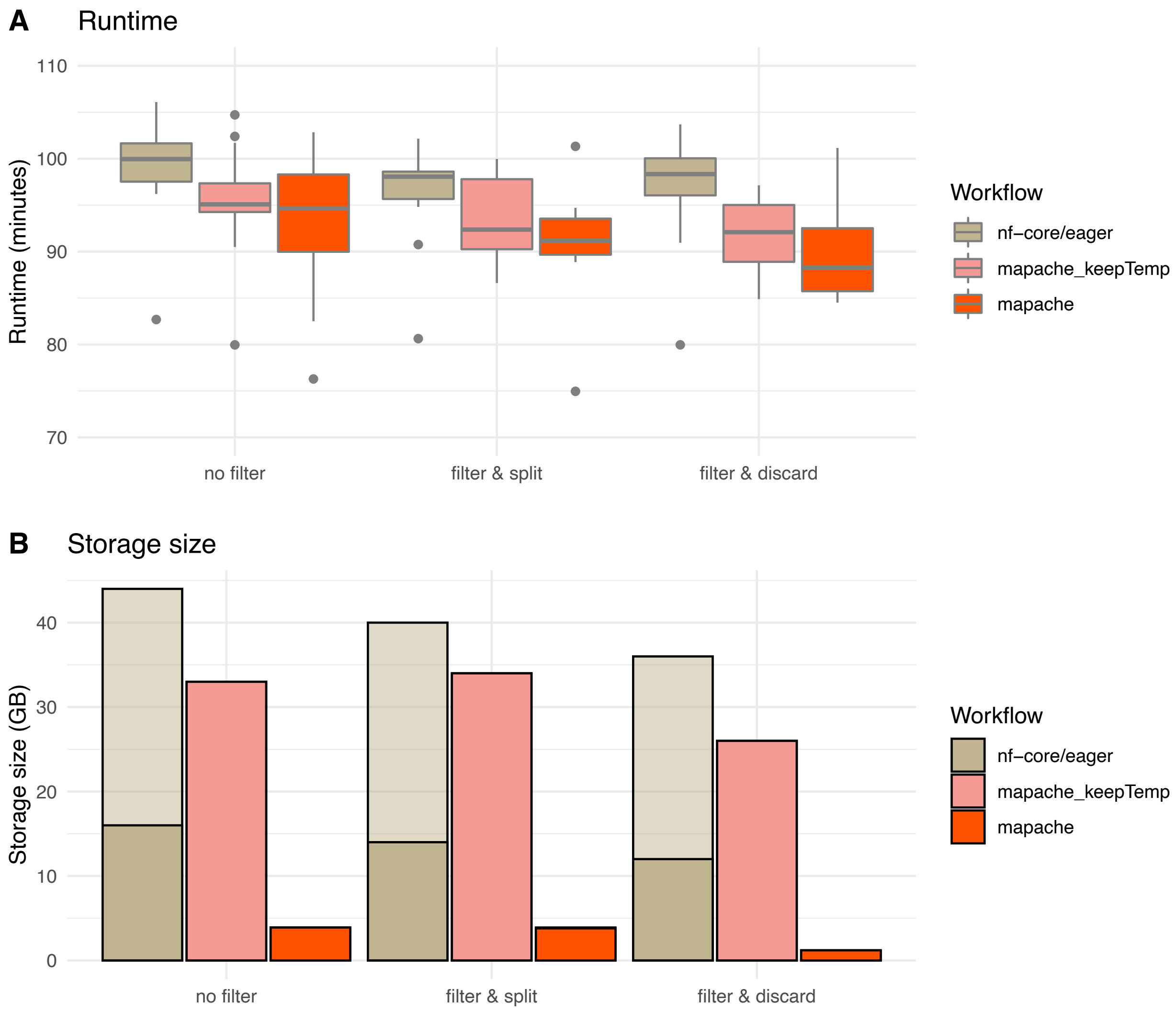

Figure 1. A) Runtime in minutes across 15 replicates. B) Storage size at the end of a run. nf-core/eager stores the data in two folders, folder 'results' contains the main output (dark color) and folder 'work' contains the intermediate files (light color).

Data availability
All data used in the benchmark are freely available.
Reference genome *Gadus morhua* (CAEA02; Tørresen, et al., 2017):

- ftp://ftp.ebi.ac.uk/pub/databases/ena/wgs/public/cae/CAEA02.fasta.gz

3 paired-end libraries of sample COD076 (Star, et al., 2017):

- ftp://ftp.sra.ebi.ac.uk/vol1/fastq/ERR194/000/ERR1943600/ERR1943600_1.fastq.gz
- ftp://ftp.sra.ebi.ac.uk/vol1/fastq/ERR194/000/ERR1943600/ERR1943600_2.fastq.gz
- ftp://ftp.sra.ebi.ac.uk/vol1/fastq/ERR194/001/ERR1943601/ERR1943601_1.fastq.gz
- ftp://ftp.sra.ebi.ac.uk/vol1/fastq/ERR194/001/ERR1943601/ERR1943601_2.fastq.gz
- ftp://ftp.sra.ebi.ac.uk/vol1/fastq/ERR194/002/ERR1943602/ERR1943602_1.fastq.gz
- ftp://ftp.sra.ebi.ac.uk/vol1/fastq/ERR194/002/ERR1943602/ERR1943602_2.fastq.gz

3 paired-end libraries of sample COD092 (Star, et al., 2017):

- ftp://ftp.sra.ebi.ac.uk/vol1/fastq/ERR194/007/ERR1943607/ERR1943607_1.fastq.gz
- ftp://ftp.sra.ebi.ac.uk/vol1/fastq/ERR194/007/ERR1943607/ERR1943607_2.fastq.gz
- ftp://ftp.sra.ebi.ac.uk/vol1/fastq/ERR194/008/ERR1943608/ERR1943608_1.fastq.gz
- ftp://ftp.sra.ebi.ac.uk/vol1/fastq/ERR194/008/ERR1943608/ERR1943608_2.fastq.gz
- ftp://ftp.sra.ebi.ac.uk/vol1/fastq/ERR194/009/ERR1943609/ERR1943609_1.fastq.gz
- ftp://ftp.sra.ebi.ac.uk/vol1/fastq/ERR194/009/ERR1943609/ERR1943609_2.fastq.gz

Configuration & command
The number of CPUs requested is 32 for mapache and Nf-core/eager.
The text in bold indicates the parameters which differ between the runs for each pipeline.

<u>Mapache (version 0.2.0)</u>

- Base config file (config/config.yaml):

```
sample_file: config/samples.tsv
genome:
  CAEA02: 'CAEA02.fasta'
indexing:
  threads: 1
adapterremoval:
  run: True
  params: '--collapse --minlength 30 --trimns --trimqualities --minquality 20 \
    --adapter1 AGATCGGAAGAGCACACGTCTGAACTCCAGTCACNNNNNNATCTCGTATGCCGTCTTCTGCTTG \
    --adapter2 AGATCGGAAGAGCGTCGTGTAGGGAAAGAGTGTAGATCTCGGTGGTCGCCGTATCATT '
  collapse_opt: 'collapse_trunc'
  threads: 4
```

```
mapping:
    mapper: 'bwa_aln'
    bwa_aln_params: '-n 0.04 -o 1 -k 2 -l 1024'
    bwa_samse_params: ''
    threads: 4
sorting:
    threads: 4
merging:
    threads: 4
damage:
    run: 'bamdamage'
    bamdamage_fraction: 5000
    bamdamage_params: '--rlength 100 --plot_length 30'
software:
    picard_jar: 'picard'
    gatk3_jar: 'GenomeAnalysisTK'
```

- Added to the config file for the ‘no filter’ run:

```
filtering:
    run: False
    threads: 4
    save_low_qual: True
```

- Added to config file for the ‘filter & split’ run:

```
filtering:
    run: True
    threads: 4
    save_low_qual: True
```

- Added to config file for the ‘filter & discard’ run:

```
filtering:
    run: True
    threads: 4
    save_low_qual: False
```

Commands

- Command for ‘mapache_keepTemp’ (note that “--notemp” in Snakemake stands for “not erasing temporary files”):

```
$ snakemake --jobs 32 --notemp;
```

- Command for the ‘mapache’ run:

```
$ snakemake -- jobs 32;
```

Nf-core/eager (version 2.4.5)

The number of CPUs is written to the config file of nextflow.

- Command for the 'no filter' run:

```
nextflow run nf-core/eager --input TSV_template.tsv --skip_preseq --fasta CAEA02.fasta \
  --clip_forward_adaptor AGATCGGAAGAGCACACGTCTGAACTCCAGTCACNNNNNNATCTCGTATGCCGTCTTCTGCTTG \
  --clip_reverse_adaptor AGATCGGAAGAGCGTCGTGTAGGGAAAGAGTGTAGATCTCGGTGGTCGCCGTATCATT \
  --min_adap_overlap 0 --mergedonly \
  --post_ar_trim_front 0 --post_ar_trim_tail 0 --post_ar_trim_front2 0 --post_ar_trim_tail2 0 \
  --bwaalnn 0.04 --bwaalno 1 --bwaalnk 2 --bwaalnl 1024 \
  --outdir ./results -profile conda;
```

- Command for the 'filter & split' run:

```
$ nextflow run nf-core/eager --input TSV_template.tsv --skip_preseq --fasta CAEA02.fasta \
  --clip_forward_adaptor AGATCGGAAGAGCACACGTCTGAACTCCAGTCACNNNNNNATCTCGTATGCCGTCTTCTGCTTG \
  --clip_reverse_adaptor AGATCGGAAGAGCGTCGTGTAGGGAAAGAGTGTAGATCTCGGTGGTCGCCGTATCATT \
  --min_adap_overlap 0 --mergedonly \
  --post_ar_trim_front 0 --post_ar_trim_tail 0 --post_ar_trim_front2 0 --post_ar_trim_tail2 0    \
  --bwaalnn 0.04 --bwaalno 1 --bwaalnk 2 --bwaalnl 1024 \
  --outdir ./results -profile conda \
  --bam_mapping_quality_threshold 30 --run_bam_filtering  --bam_unmapped_type bam ;
```

- Command for the 'filter & discard' run:

```
$ nextflow run nf-core/eager --input TSV_template.tsv --skip_preseq --fasta CAEA02.fasta \
  --clip_forward_adaptor AGATCGGAAGAGCACACGTCTGAACTCCAGTCACNNNNNNATCTCGTATGCCGTCTTCTGCTTG \
  --clip_reverse_adaptor AGATCGGAAGAGCGTCGTGTAGGGAAAGAGTGTAGATCTCGGTGGTCGCCGTATCATT \
  --min_adap_overlap 0 --mergedonly \
  --post_ar_trim_front 0 --post_ar_trim_tail 0 --post_ar_trim_front2 0 --post_ar_trim_tail2 0 \
  --bwaalnn 0.04 --bwaalno 1 --bwaalnk 2 --bwaalnl 1024 \
  --outdir ./results -profile conda \
  --bam_mapping_quality_threshold 30 --run_bam_filtering --bam_unmapped_type discard ;
```

References

Picard tools. In, *Broad Institute, GitHub repository*. http://broadinstitute.github.io/picard: Broad Institute; 2019.

Danecek, P., *et al.* Twelve years of SAMtools and BCFtools. *GigaScience* 2021;10(2).

Li, H. and Durbin, R. Fast and accurate long-read alignment with Burrows-Wheeler transform. *Bioinformatics* 2010;26(5):589-595.

Malaspinas, A.S., *et al.* bammds: a tool for assessing the ancestry of low-depth whole-genome data using multidimensional scaling (MDS). *Bioinformatics* 2014;30(20):2962-2964.

Neukamm, J., Peltzer, A. and Nieselt, K. DamageProfiler: fast damage pattern calculation for ancient DNA. *Bioinformatics* 2021;37(20):3652-3653.

Schubert, M.*, et al.* Characterization of ancient and modern genomes by SNP detection and phylogenomic and metagenomic analysis using PALEOMIX. *Nat Protoc* 2014;9(5):1056-1082.

Schubert, M., Lindgreen, S. and Orlando, L. AdapterRemoval v2: rapid adapter trimming, identification, and read merging. *BMC Res Notes* 2016;9:88.

Star, B.*, et al.* Ancient DNA reveals the Arctic origin of Viking Age cod from Haithabu, Germany. *Proceedings of the National Academy of Sciences of the United States of America* 2017;114(34):9152-9157.

Tørresen, O.K.*, et al.* An improved genome assembly uncovers prolific tandem repeats in Atlantic cod. *BMC Genomics* 2017;18(1).

Yates, J.A.F.*, et al.* Reproducible, portable, and efficient ancient genome reconstruction with nf-core/eager. *PeerJ* 2021;9.
